# Supplementary material for: Seminoma and Embryonal Carcinoma Footprints Identified by Analysis of Integrated Genome-Wide Epigenetic and Expression Profiles of Germ Cell Cancer Cell Lines
Source: PLoS One. 2014 Jun 2;9(6):e98330. doi: 10.1371/journal.pone.0098330 (PMC4041891; doi:10.1371/journal.pone.0098330)
Supplement: File S1 — ZIP file containing DMRforPairs output for significant regions. Please start from the html files. (ZIP) [file pone.0098330.s008.zip › figures/33995.pdf]

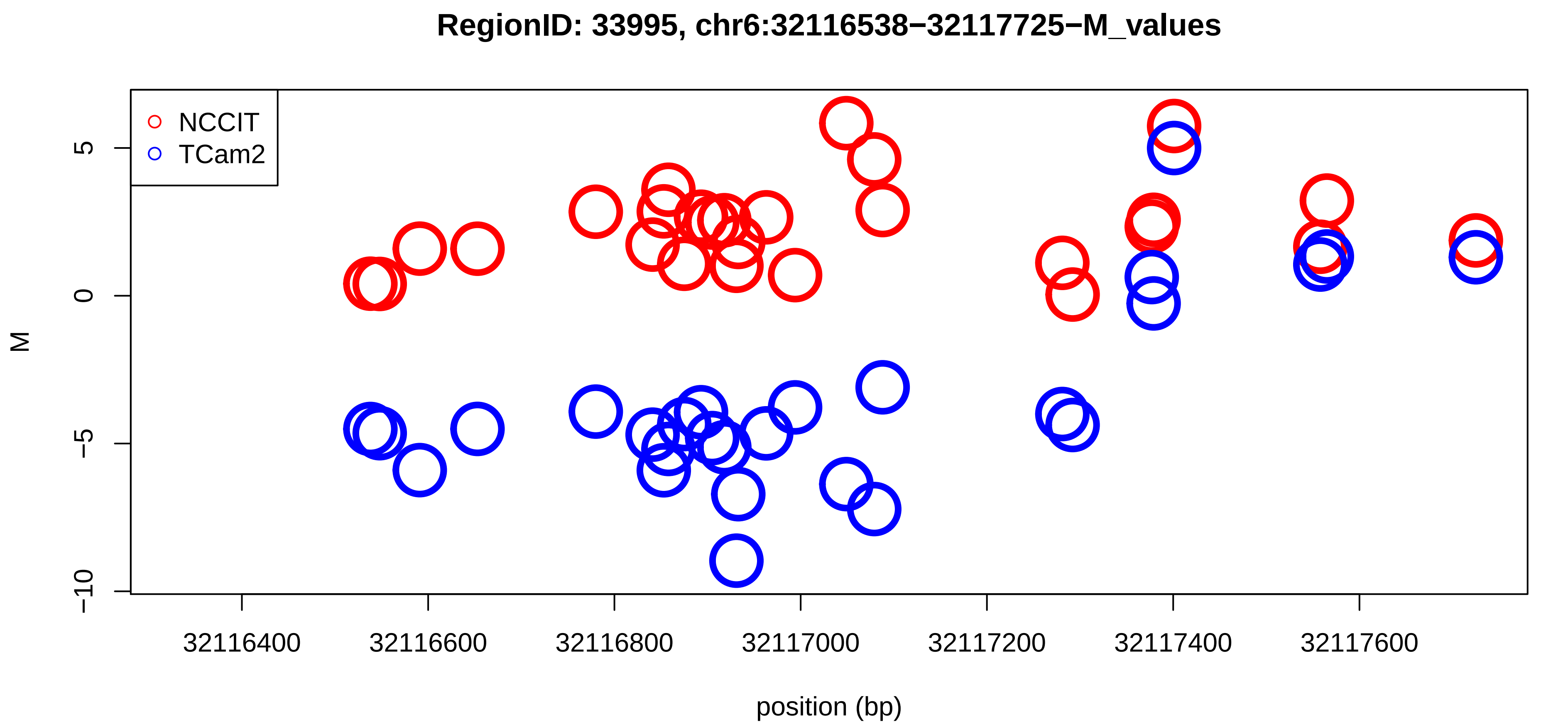
RegionID: 33995, chr6:32116538−32117725−M_values
NCCIT
TCam2
M
5
0
−5
−10
32116400
32116600
32116800
32117000
32117200
32117400
32117600
position (bp)

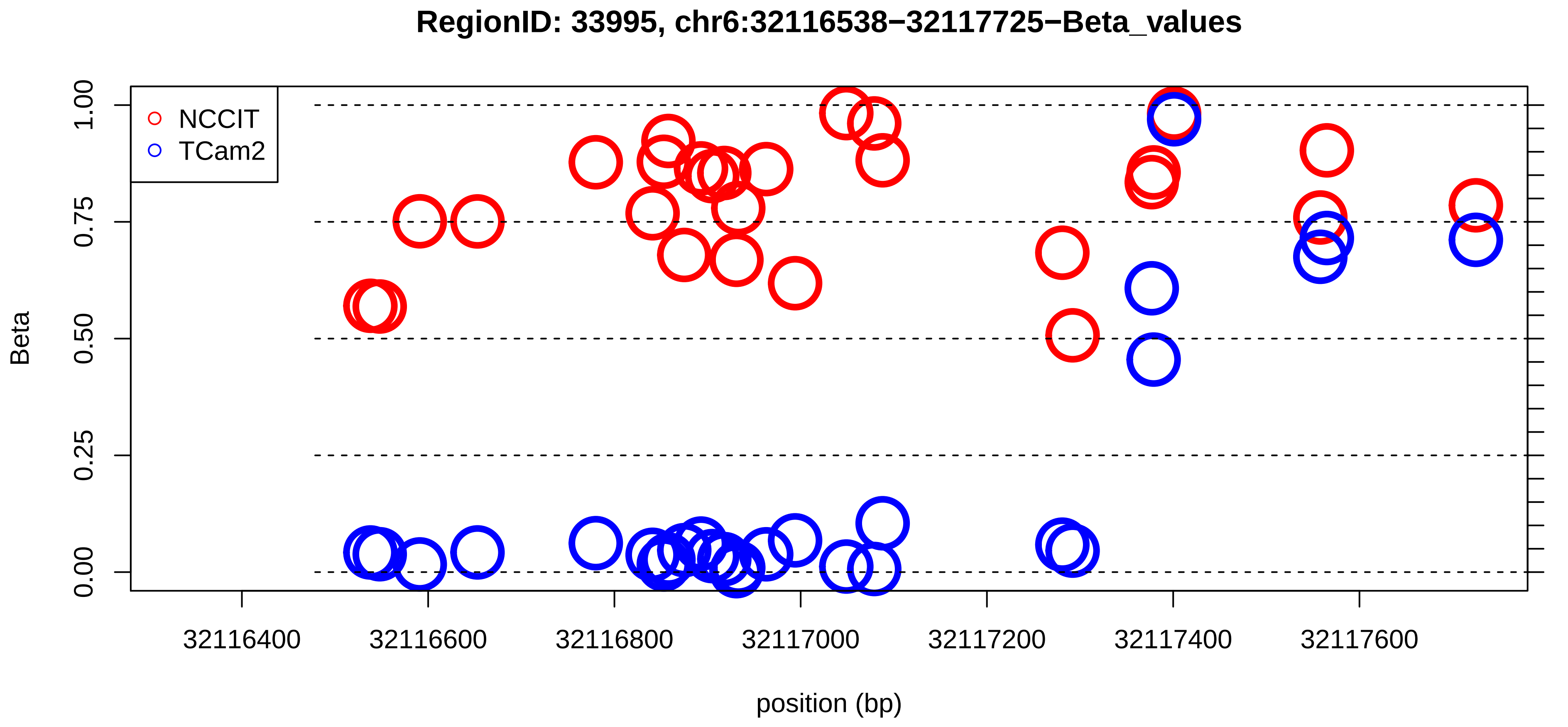
RegionID: 33995, chr6:32116538−32117725−Beta_values
NCCIT
TCam2
Beta
1.00
0.75
0.50
0.25
0.00
32116400
32116600
32116800
32117000
32117200
32117400
32117600
position (bp)

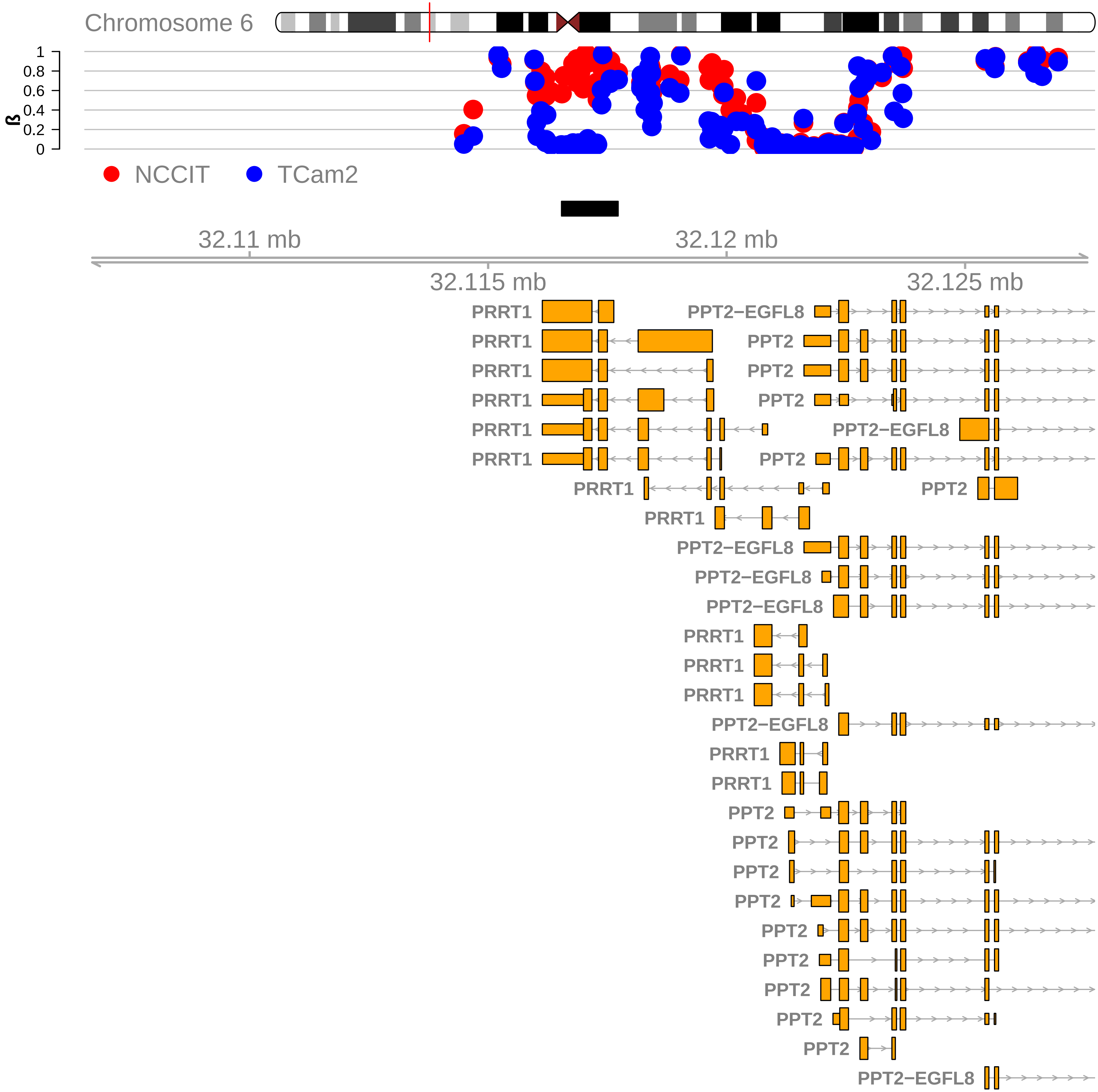
Chromosome 6
β
1
0.8
0.6
0.4
0.2
0
NCCIT
TCam2
32.11 mb
32.12 mb
32.115 mb
32.125 mb
PRRT1
PPT2−EGFL8
PPT2
